# Protective immune response in rainbow trout (*Oncorhynchus mykiss*) against the parasitic nematode *Anisakis simplex*

**DOI:** 10.3389/fimmu.2025.1646450

**Published:** 2025-08-20

**Authors:** Kaan Kumas, Maja Hauptmann Andersen, Rzgar Jaafar, Cyril Henard, Per Walter Kania, Kurt Buchmann

**Affiliations:** Laboratory of Aquatic Pathobiology, Department of Veterinary and Animal Sciences, Faculty of Health and Medical Sciences, University of Copenhagen, Frederiksberg, Denmark

**Keywords:** nematodes, fish, immunity, protection, antigens

## Abstract

**Introduction:**

Parasitic nematodes are prevalent in fish populations. The parasites are pathogenic but depress host responses, which limit clearance of the pathogens from the invasion sites. We hypothesized that one of several control strategies, which could augment protection, is immunization of the fish host with parasite antigens prior to live pathogen exposure.

**Methods:**

We used rainbow trout *Oncorhynchus mykiss* as a host model and third stage larvae (L3) *Anisakis simplex* (Nematoda, Ascaridoidea, Anisakidae) as pathogen model. We used a total of 120 fish and immunized 40 of the fish with a homogenate (adjuvanted) of parasite larvae (i.p. injection), 40 fish received adjuvant only and 40 PBS. Following 38 days (d) half of the fish in each group were exposed to infection with live worms (oral administration), and after an additional 25 d the infection success was evaluated together with antibody responses in the different groups.

**Results:**

Injection of *A. simplex* L3 antigens induced a series of adaptive and innate host responses. ELISA and Western blot analyses indicated specific IgM reactions in immunized trout against worm antigens with molecular weights (MW) of approximately 39, 103 and 119 kilodalton (kDa). Fish immunized and subsequently infected with live larvae reacted to those three and six additional antigens with MW approximately 61, 73, 84, 152, 186 and 277 kDa. The immunized fish showed a significantly lower worm burden following exposure to live parasite larvae (when compared to naïve fish), but no full protection was achieved. Expression analyses of both adaptive and innate immune genes in fish showed a general down-regulation following infection.

**Discussion:**

Prior immunization with *A. simplex* L3 homogenate induced a strong antibody response, but the protection was incomplete. It was noteworthy that an infection period (25 d) with live parasites merely induced an insignificant antibody response. It may be explained by the immunosuppressive compounds released by live worm larvae. With the aim of increasing the protective response, we suggest in future immunization experiments to target immunosuppressive worm antigens by immunizing the host organisms with excretory/secretory (ES) proteins and extracellular particles from *A. simplex* L3.

## 1 Introduction

During evolution endoparasites evolved a range of mechanisms to locate hosts, penetrate their external surfaces, survive in internal tissues and reproduce in the target organism (1). Some parasites, such as ascarid nematodes, attain a considerable size compared to virus and bacteria, which may be one of the reasons they are not readily engulfed, degraded and eliminated by host immune cells, whereby they may remain in various body compartments for extended time periods (2, 3). The measurement scale reflects this as size of helminths are often measured by millimeter (mm) or centimeter (cm), whereas bacteria are measured in microns and virus in nanometer (nm). In addition, parasites possess mechanisms allowing them to evade innate immune responses (4), which may delay elimination of the invader from the host organism. The immune evasion mechanisms include a wide range of factors (enzymes, enzyme inhibitors, antiproteases, host defence molecules, host homologs, miRNAs), which are masking parasite antigens, skewing reactions towards tolerance, blocking immune pathways and interfering with central immune effector molecules (4). However, field studies on age and size related *Anisakis* infection of Atlantic mackerel *Scomber scombrus* have indicated that this species is able to clear infections over time, even in a highly infective environment (5). We therefore hypothesize that it is possible to partially overcome the immune evasion strategy by immunization of fish with *A. simplex* L3 antigens before infection with live worms. During recent years several studies have documented that various non-teleost vertebrates achieve at least some protection against helminth parasites following vaccination with parasite antigens. This was reported for various species of hookworm nematodes (6–8), ascarid nematodes (9–13), trematodes (14) and cestodes (15). Aiming at testing this hypothesis we established a host-parasite model with rainbow trout *Oncorhynchus mykiss* as host, and third stage larvae (L3) of the nematode *Anisakis simplex* as parasite. We immunized the fish by intraperitoneal injection of a worm homogenate and subsequently measured the antibody response, immune gene expression and any protection conferred. This was done by experimental infection using live worm larvae. Salmonid fishes are generally susceptible to *A. simplex* third stage larvae. Atlantic salmon *Salmo salar* have been found naturally infected in the wild (16–19), and both brown trout *S. trutta* and rainbow trout can easily be experimentally infected by oral administration of live larvae (20–23). A well-suited source of nematode larvae for experimental infection studies is freshly captured Atlantic herring *Clupea harengus* from the North Sea, which carry significant larval burdens in their body cavities (24, 25). A suitable fish host for controlled experimental infection is rainbow trout, which can be obtained as naïve and pathogen-free fish from biosecured recirculated systems (26).

## 2 Materials and methods

### 2.1 Ethics

The experiment was conducted under license 2024-15-0201–01697 issued by the Experimental Animal Inspectorate (Denmark) and therefore review by the Animal Ethical Institutional Review Board of the University of Copenhagen was exempted.

### 2.2 Experimental design

The overall aim was to elucidate whether prior immunization of rainbow trout with parasite immunogens would confer some protective immunity to infections with live nematode parasites. The experiment was performed in duplicate, resulting in an overall setup of 120 fish (2x60). Within each replicate 20 fish were kept as untreated control (injected with PBS only), 20 fish were injected with the adjuvant, and 20 fish were immunized by injection of immunogen with adjuvant. Five weeks after immunization, half of the fish in each of these groups were exposed to infection with live nematode larvae, whereafter the fish were followed for an additional 25 days (d). This doubled the number of study groups (infected and non-infected subgroups of the PBS injected control fish, adjuvant treated fish and immunized fish). The fish were kept in two common garden set-up systems, each with six groups (containing 10 fish in each group), which guaranteed that all groups were exposed to the same external environment. The groups were differentiated by fin cut (see below).

### 2.3 Fish

Rainbow trout (all females) originated from a certified virus-free stock (Hallesø trout farm, Jutland, Denmark). Eyed eggs were transported (airfreight at cooled conditions) to the pathogen-free recirculating aquaculture system (RAS) facility (Aqua Baltic, Nexø, Bornholm, Denmark). Following arrival, eggs were disinfected (Buffodine, Biomar, Denmark) and finally placed in hatching trays (26). Egg hatched 14–21 d later, whereafter the fish were reared to the juvenile stage before being transferred (3 h car transport in oxygenated cooled water) to the infection facility at the University of Copenhagen. The 120 specimens of rainbow trout used for the study were selected for size uniformity (mean body weight 8 gram (g), mean body length 9 centimeter (cm)).

### 2.4 Tagging of fish groups

In order to differentiate the experimental groups, the fish, assigned to different experimental groups, were differentially tagged by fin cuts. Briefly, following anaesthesia (by immersion into a solution of 40 mg/L tricaine methane sulfonate, MS-222, Sigma–Aldrich, Denmark) a minor part of the tail or pelvic fins were removed with a surgical scissor. The upper part of the caudal tail was cut in the immunized group, a lower part of the caudal tail was cut in the adjuvant group, and a middle part of the caudal tail was cut in the PBS control group. Following exposure to live parasites, differentiation of infected and non-infected fish groups was achieved by cutting a minor part of the right pelvic fin and left pelvic fin, respectively.

### 2.5 Fish keeping at infection facility

Upon arrival, fish were acclimatized in two tanks (volume 530 L each) for two weeks prior to experimental procedures. The tanks (volume 530 L each) were filled with tap water (pH 7.4, Calcium carbonate 450 mg/L) (Frederiksberg municipality), equipped with internal bio-filters (Eheim, Pickup 200, Germany) for recirculation and temperature kept at 19°C (thermostat regulated room). They were constantly aerated (double inflow) securing sufficient oxygen saturation, and 20% daily water exchange secured low ammonia, nitrite and nitrate levels as measured by water quality strips twice a day (Tetra, Melle, Germany). The fish were fed with commercial trout feed (Inicio, Biomar, Denmark) at a 2% biomass feeding rate per day.

### 2.6 Live parasites for immunogen preparation and experimental infection

Live third-stage *A. simplex* larvae (L3) were isolated on two occasions from Atlantic herring *Clupea harengus* (caught in ICES division A4, North Sea) and kept in Petri dishes with tap water until they actively had escaped their encapsulation by host cells. For the immunogen preparation (to be used for fish immunization and coating of ELISA plates) we preserved 250 larvae in 96% ethanol at 4°C (24 h), whereafter they were subjected to homogenization (see below). Live worm larvae for the experimental infection were isolated at a later occasion (two days before infection procedure) and kept in Petri dishes containing tap water at room temperature (RT). Activity of the nematodes was noted according to (27) and only active nematodes (a total 600) without encapsulation were selected and used for the infection.

### 2.7 Immunogen production

A crude extract was prepared by homogenization of a total of 250 specimens of 96% ethanol preserved *A. simplex* L3. Larvae were washed twice in phosphate-buffered saline (PBS) with penicillin-streptomycin solution (Sigma-Aldrich, Denmark). Worms were then cut into pieces with a scalpel and transferred to a 25 mL sterile Falcon tube containing 12mL PBS with 0.1% Triton X-100 (Calbiochem, MA, USA) solution and sonicated on ice (Branson Ultrasonics, USA). The homogenate was centrifuged at 9,000 revolutions per minute (rpm) for 1 hour (h) at 4°C, whereafter the supernatant was aliquoted into Eppendorf tubes and stored at -20°C until use. Protein concentration of the supernatant was determined by the bicinchoninic acid method (PierceTM BCA Protein Assay Kit, Thermo Scientific, Denmark) and read at a wavelength of 562 nanometer (nm) using a spectrophotometer (Epoch, BioTek, Winooski VT, USA).

### 2.8 Immunization

The fish were anaesthetized by immersion into a solution of 40 mg/L tricaine methane sulfonate (MS-222, Sigma–Aldrich, Denmark). Intraperitoneal (IP) injections into the body cavity of fish (three experimental groups) were performed: 1) Immunized group: fish were injected with 0.2 mL (1:1 ratio) of immunogen (supernatant of the worm protein homogenate) (200 µg/per fish) and adjuvant (Freund’s incomplete adjuvant, cat. no. F5506, Sigma–Aldrich, Denmark), 2) Adjuvant control group: Fish were injected with 0.2 mL (1:1 ratio) of PBS and adjuvant, 3) PBS control group: Fish were injected with 0.2 mL PBS only. Freund’s incomplete adjuvant is a mineral oil, and when mixed with the antigen solution (water-in-oil), it secures a persisting antigen exposure at the injection site.

### 2.9 Infection with live larvae

Five weeks post-immunization rainbow trout (immunized, adjuvant injected control, PBS injected control) were challenged (experimentally exposed) to live *A. simplex* L3, which were isolated from freshly caught herring sourced from a commercial vessel (ICES 4a area of the North Sea) as described above. Half of the fish in all groups (60 fish) were infected with 10 live *A. simplex* L3 larvae per fish via gastric installation using forceps. The infection procedure included 1) Anaesthesia by immersion into a MS222 solution (40 mg/L) (Sigma–Aldrich, Denmark), 2) Transfer of nematode larvae by use of a soft pair of forceps directly into the ventricle. Following infection, each fish was placed in an aquarium for 30 min for observation ensuring fitness and that no worm larvae left the fish. Non-infected fish were treated similarly (anaesthesia, gastric installation) but without the use of live larvae. This secured similar handling of fish (apart from the infection). Following parasite exposure fish were kept for 25 d, whereafter samples were taken and the experiment terminated.

### 2.10 Infection level measurement

Sampling was performed 25 days after the challenge. All fish were euthanized (MS222 immersion 300 mg/L). Each fish was weighed and measured (final mean weight 24 g and mean total length 13 cm). Samples were taken for Enzyme-linked immunosorbent assay, Western Blot (WB) (blood) and immune gene expression analysis (spleen and liver). In order to precisely count the number of nematodes in the fish they were immediately frozen in individual plastic bags at –20°C until parasitological examination. Following thawing visual inspection under a dissection microscope (Leica S6E, Germany) was conducted to detect the presence of nematodes in the body cavity, mesenteries, stomach, pyloric caeca, intestine, liver, heart, swim bladder, spleen, and musculature. After dissection, all organs were subjected to pepsin/HCl digestion to ensure that all nematodes were recovered (28). The flesh was examined either by UV detection or pepsin digestion (29). The location and number of *A. simplex* L3 were noted. The collected nematode larvae were then preserved in 96% ethanol for morphological identification and molecular analysis.

### 2.11 Melano-macrophage aggregation

Melano-macrophage aggregations were used as a rough indication of innate cellular reactions. They were seen as black cell aggregations/patches (1–4 mm in diameter) located in the body cavity (mesenteries, organs). The extent of these was recorded and semi-quantitatively scored on a scale from 0 to 3: 0 (no black spots), 1 (1–2 spots), 2 (3–5 spots) and 3 (>5 spots).

### 2.12 Blood sampling

Immediately upon euthanasia, the fish blood was extracted by caudal vein puncture using heparinized syringes (volume 1 mL). Blood samples were kept on ice until centrifugation (Hettich 320 R, Tuttingen, Germany) for 5 min at 4000 rpm, whereafter plasma was recovered and stored at – 20°C until use.

### 2.13 Sampling of immune organs for gene expression analysis

Following blood sampling, necropsy was performed and liver and spleen samples were immediately transferred to RNAlater for subsequent gene expression analyses. These samples were kept at 4°C for 24 h and then stored at -20°C until use (RNA purification).

### 2.14 Antibody response measurement

An indirect Enzyme-linked immunosorbent assay (ELISA) was conducted to assess the immunoglobulin (IgM) response in rainbow trout plasma samples (30). MaxiSorp™ ELISA plates (VWR, Denmark) were coated (at 4°C overnight on shaker, 50 rpm) with *A. simplex* antigens (the immunogen preparation diluted to 3.4 μg protein/mL in bicarbonate coating buffer (Sigma-Aldrich, Denmark). The plates were washed with PBST (PBS containing 0.1% Tween 20 (Sigma-Aldrich, Denmark)), then blocked with 2% Bovine Serum Albumin (BSA) (Thermo Scientific, Denmark) in PBS and stored at -20°C until use. The subsequent steps were performed at room temperature (RT). To determine working dilutions in different groups, fish plasma samples were serially diluted (1:10 to 1:10.000.000) and tested. Minimal interference of unspecific binding was considered and the 1:5000 plasma dilution was then selected for subsequent measurements. The diluted plasma samples were added to each well, and the plates were incubated overnight. After washing (3x PBST), the secondary antibody (mouse anti-salmonid Ig, AbD Serotec, Germany) was added and incubated for 1 h followed by a wash (3x PBST). Then the tertiary antibody (HRP-conjugated rabbit anti-mouse IgG, AbD Serotec, Germany, diluted 1:500) was added for 1h incubation. After a last wash, TMB (BioRad, Denmark) substrate was added, incubated for 15 minutes, whereafter the reaction was stopped with 1 N HCl. Absorbance was measured (Spectrophotometer Epoch, BioTek, Winooski VT, USA) at 450 nm.

### 2.15 SDS-PAGE analysis

SDS-PAGE (reduced conditions) was performed to assess the size of the *A. simplex* L3 proteins in the immunogen (supernatant of the homogenate), and the Western Blot was done to determine if the fish antibodies reacted to any specific parasite proteins. Non-diluted and diluted series of the sample were prepared to determine the best gel visualization. We prepared samples with NuPAGE™ LDS sample buffer (4X) and Reducing Agent (10X) at 70°C for 10 min according to the manufacturer’s instructions (Invitrogen, Denmark). The samples were applied on pre-casted NuPAGE gels (4-12% NuPAGE Bis-Tris gels, Invitrogen, Denmark) and run in a XCell SureLock™ electrophoresis cell (Invitrogen, Denmark) containing 1X SDS Running buffer, which was prepared by diluting 20X NuPAGE™ MES running buffer (Invitrogen, Denmark) with distilled water. NuPAGE™ Antioxidant (Invitrogen, Denmark) was added for reduced samples and the gels were run at 100 voltage (V) for 80 min. Protein bands were visualized by SimplyBlue™ SafeStain (Invitrogen, Denmark).

### 2.16 Western blot analysis

The Western Blot (WB) procedure was conducted as specified by (31) with some modifications. In brief, separated proteins (SDS-PAGE, reduced conditions, without staining) were transferred onto a 0.22µm pore size Polyvinylidene Fluoride (PVDF) Transfer Membrane (Mikrolab-Frisinette A/S, Denmark) in an XCell II Blot Module running at 30V for 90min. The membrane was blocked with 2% BSA and incubated overnight at 4°C with fish plasma as primary antibody (diluted 1:50). As secondary antibody we used the mouse anti-salmonid Ig (AbD Serotec, Germany) (1h incubation at RT). HRP (horseradish peroxidase) conjugated rabbit anti-mouse IgG (AbD Serotec, Germany, diluted 1:500) was used as tertiary antibody (1h incubation at RT). Visualization of the reactivity was achieved by adding substrate 3,3′-Diaminobenzidine (SIGMAFAST™, Sigma-Aldrich, Denmark), which resulted in a brown precipitate. PBST was used to wash the membrane between each step.

### 2.17 RNA extraction and cDNA production

From each of the 6 experimental groups, liver and spleen from 20 fish (in total 240 samples) were isolated and subjected to RNA extraction and cDNA synthesis, which were performed according to standard procedures (34). In brief, homogenization (Tissue-lyser II, Qiagen, Denmark) samples were performed in 2-mercaptoethanol homogenization buffer. To ensure proper release of nucleic acids from liver samples, pre-treatment with Proteinase K (Sigma-Aldrich, Denmark) were performed. The GeneEluteTM mammalian RNA kit (Sigma-Aldrich, Denmark) was used to purify the RNA. Genomic DNA was removed by DNase1 treatment, concentration of RNA was measured using a Nanodrop 2000 spectrophotometer (Thermo Scientific, Denmark) and quality was assessed by electrophoresis on a 2% agarose gel stained with ethidium bromide (Invitrogen, Denmark). The RNA was preserved at -80°C until use. The cDNA synthesis was performed using the TaqMan^®^ Reverse Transcription Kit (Thermo Fisher Scientific, Denmark) in a 20 µl reaction with 1000 ng of RNA with Oligo d(T)16 primer and 1µL reverse transcription reagent. Controls without the enzyme transcriptase were included.

### 2.18 Quantitative realtime gene expression

qPCR was performed using the TaqMan^®^ probes A mix was prepared containing Brilliant III Ultra-Fast Master Mix (AH diagnostics AS, Denmark) and target gene primers (presented in **Supplementary Table 1**). To investigate expression of relevant immune genes in rainbow trout the following 31 genes were targeted: Genes encoding interleukins (IL-1β, IL-2, IL-4/13α, IL-6, IL-8, IL-10, IL-12, IL-17A/F2, IL-17C1, IL-17C2, IL-22), type II interferon (IFNγ), transforming growth factor β (TGFβ), tumor necrosis factor α (TNFα). Also genes encoding effector molecules such as serum amyloid A (SAA), complement factor 3 (C3), immunoglobulins (IgM, IgDm, IgT, IgDs), cathelicidins (Cath-1, Cath-2), lysozyme, and cellular makers including T cell receptor β (TCRβ), major histocompatibility complex molecules (MHC I, MHC II), T cell markers (CD4, CD8). Housekeeping genes functioning as reference genes were ARP, B-actin, and ELF-1α (32–34) by using the average of the Cq values based on NormFinder (35). All qPCR assays were performed in the AriaMx™ real-time PCR system (AH diagnostics AS, Denmark) using the following conditions: 95°C for 3 min, 40 cycles of 95°C for 5 s and 60°C for 15 s, followed by endpoint measurement. The qPCR results were analyzed using the simplified 2^-ΔΔCq^ method (36) as all the qPCR assays had efficiencies within ±5%. Significant gene regulation was only considered when regulation was at least ±2 and one-way ANOVA with Šídák’s multiple comparisons test resulted in *P<0.05*.

### 2.19 Morphological identification of nematodes

Subsamples (total of 10) of nematode larvae, used for both immunogen production and for infection, were selected. The individual worms were divided into anterior, middle and caudal parts. Morphological analyses were performed with anterior and caudal parts of the parasite, whereas molecular analyses were carried out with the middle parts. Morphological identification to the genus level was performed on parasites conserved in 96% ethanol. Parasites were transferred individually into heavy glass beaker (staining cups) containing lactic acid and kept until nematode part parts were clear, whereafter they were placed on microscope slide with glycerin-gelatine (pre-heated to 55°C) (37). The refraction index of lactic acid and glycerine secured that surface structures of the worms became transparent and organs became visible. Examination was carried out under light microscope with phase-contrast (Leica DM5000B, Germany) at different magnifications (X100-400).

### 2.20 Molecular identification of nematodes

The middle part of aseptically cut nematodes (total 10), preserved in 96% ethanol and kept at 4°C, were used for molecular identification. Molecular identification was carried out by PCR and subsequent sequencing by targeting mitochondrial DNA (mtDNA) and ribosomal DNA (rDNA). Purification of the genomic DNA was performed using QIAamp DNA Mini Kit (cat. No. 61306, Qiagen, Denmark) according to manufacturer’s instructions except that the elution buffer volume was 50µl instead of 200µl. A Thermal Cycler BioRad T100 (Bio-Rad Laboratories, Denmark) was used to conduct the PCR reaction. The reaction volumes were total 60 µl composed of 6 μL of 10× Reaction buffer, 1.8 μL of 50 mM MgCl2, 0.6 μL of DNA Polymerase, (all three BIOTAQ DNA Polymerase, Saveen & Werner ApS, Jyllinge, Denmark), 6µl of 10 mM dNTPmix (Applied Biosystems™ GeneAmp™ dNTP Blend (100 mM) Fisher Scientific, Slangerup, Denmark), 6 μL of forward and reverse primers (Tag Copenhagen, Frederiksberg, Denmark) (both 10 mM) 5 μL of the sample and finally 28.6 μL of RNase-free water (Fisher Scientific, Denmark). The internal transcribed spacer region (ITS) was targeted by using the forward primer PDG_18S_F5 (5′-CGATAACGAACGAGACTC-3′) (38) and the reverse primer NC2 (5′-TTAGTTTCTTTTCCTCCGCT-3′) (39). Forward primer 211F_alt (5′-TTTTCTAAGTTATATAGATTGRTTTYAT-3′) (37) and reverse primer 210R (5′-CACCAACTCTTAAAATTATC-3′) were used to target mtDNA (40). The PCR conditions for the ITS and the mitochondrial gene *cox*2 were similar with some modifications at elongation and post-elongation step. PCR for ITS region was conducted by one cycle of pre-denaturation at 95 °C for 5 min, 40 amplification cycles at 95 °C for 30s, annealing was performed at 54°C for 30s and elongating at 72 °C for 90 s, followed by a post-elongation step at 95°C for 7min. PCR conditions for the *cox2* gene amplification were the same except for the elongation time (60 s) and post-elongation (5 min). Visualization of the PCR products were performed by 2% agarose gel electrophoresis. PCR products were purified according to manufacturer’s instructions using Illustra™ GFX™PCR DNA and Gel Band Purification Kit (VWR International A/S, Søborg, Denmark) and finally sequenced at Macrogen Europe (Netherlands). Sequence analyses were performed by use of the software CLC-Main Workbench v20.0.4 (QIAGEN, Hvidovre, Denmark).

### 2.21 Phylogenetic analysis

The phylogenetic analysis was performed using the software CLC Main Workbench Version 20.0.4 (Qiagen, Denmark). In the case of the rDNA, the flanking 18S and 28S were used during the construction of the alignment using Clustall W. After minor editing, the 18S and 28S were removed, thus the ITS (internal transcribed spacer) region consisting of ITS1 the 5.8 rRNA and ITS2 remained for further analysis. Likewise, the complete achieved sequences of the mtDNA gene *cox2* were used for alignment (Clustall W), but only the part between the forward primer 211F and the reverse primer 210R was used for further analysis. In both cases, model testing suggested that GTR+G+T was the best evolutionary model. Two threes were constructed by the means of maximum likelihood phylogeny using a UPGMA initial tree and performing 1000 bootstraps. The two trees are presented as phylograms in **Supplementary Figure 2**.

### 2.22 Statistics

Infection levels (total number of worms, prevalence, mean intensity and abundance) of the fish challenged with parasites were calculated according to (41). If normal distribution was established using the Kolmogorov-Smirnov test, the parametric one-way ANOVA with Šídák’s multiple comparisons tests and the Pearson r correlation test were performed. If data did not follow a Normal distribution the nonparametric Mann-Whitney test, Kruskal-Wallis with Dunn’s multiple comparison, and Spearman correlation test were used. All tests were conducted by GraphPad Prism 10.4.2. (USA). A general probability level of 5% was applied (*P<0.05*).

## 3 Results

### 3.1 Infection levels

**Figure 1:**
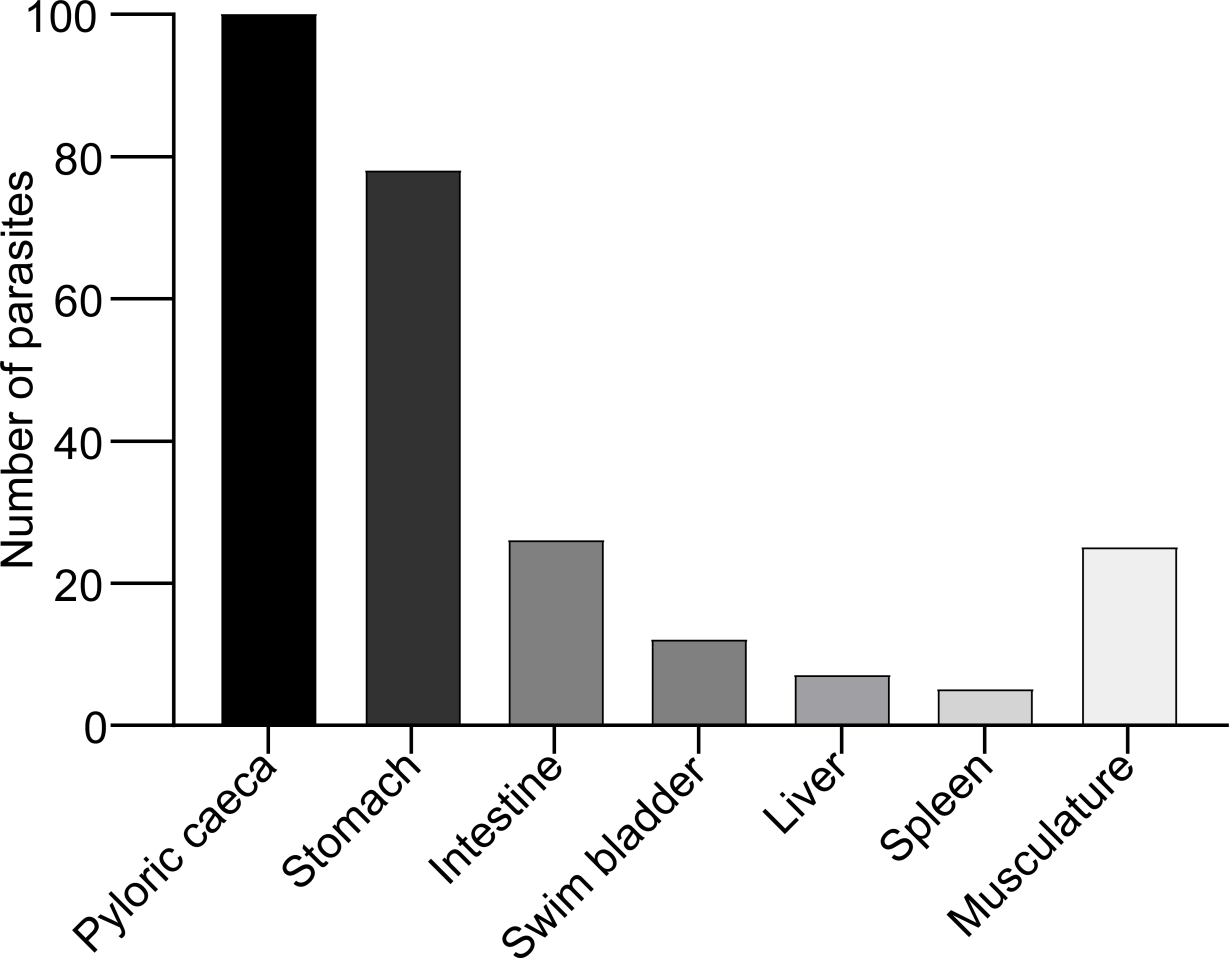
Location of *A. simplex* (L3) along the body cavity organs and musculature in rainbow trout (data obtained from all experimental fish infected with *A. simplex*).

When fish were examined at 25 days (d) following experimental infection with *A. simplex* L3 a total of 70 nematodes were recovered from the immunized group, 92 nematodes were recovered from adjuvant group and 91 nematodes were recovered from the PBS injected (control) group. The prevalence of the infection for immunized, adjuvant and PBS control group were 90%, 100% and 100% respectively. The mean intensities were 3.88, 4.6, 4.55 and abundances were 3.5, 4.6 and 4.55 for the immunized, adjuvant and PBS groups, respectively. The nematode load in the immunized group was significantly different from both adjuvant (*P:0.03*) and the PBS control group (*P:0.04*) (one tailed Mann-Whitney, *P< 0.05*). The majority of nematodes (90%) were located along the mesenteries of body cavity organs (pyloric caeca, stomach, spleen, intestine, liver, swimming bladder) whereas only 10% of worms were found in the musculature (**Figure 1**). The larvae in the body cavity were mainly located on pyloric caeca (39.52%), whereas (30.83%) were found in association with the stomach.

### 3.2 Melano-macrophage aggregation

Melano-macrophages aggregations were visible as black patches covering parasites and organs in the body cavity organs. The cell layers were only observed in the adjuvant (n=41) and immunized infected group (n=45) following exposure to live nematodes. This response was not seen in the PBS injected group (zero black patches). Macrophage aggregation levels were graded on a scale from 0 to 3, and when a two-tailed Pearson correlation test was performed between the nematode count and macrophage levels, a significant negative correlation was found (*r= -0.4607; P<0.05*) only in the immunized group. No correlation was found in the other groups.

### 3.3 Specific antibody levels

**Figure 2:**
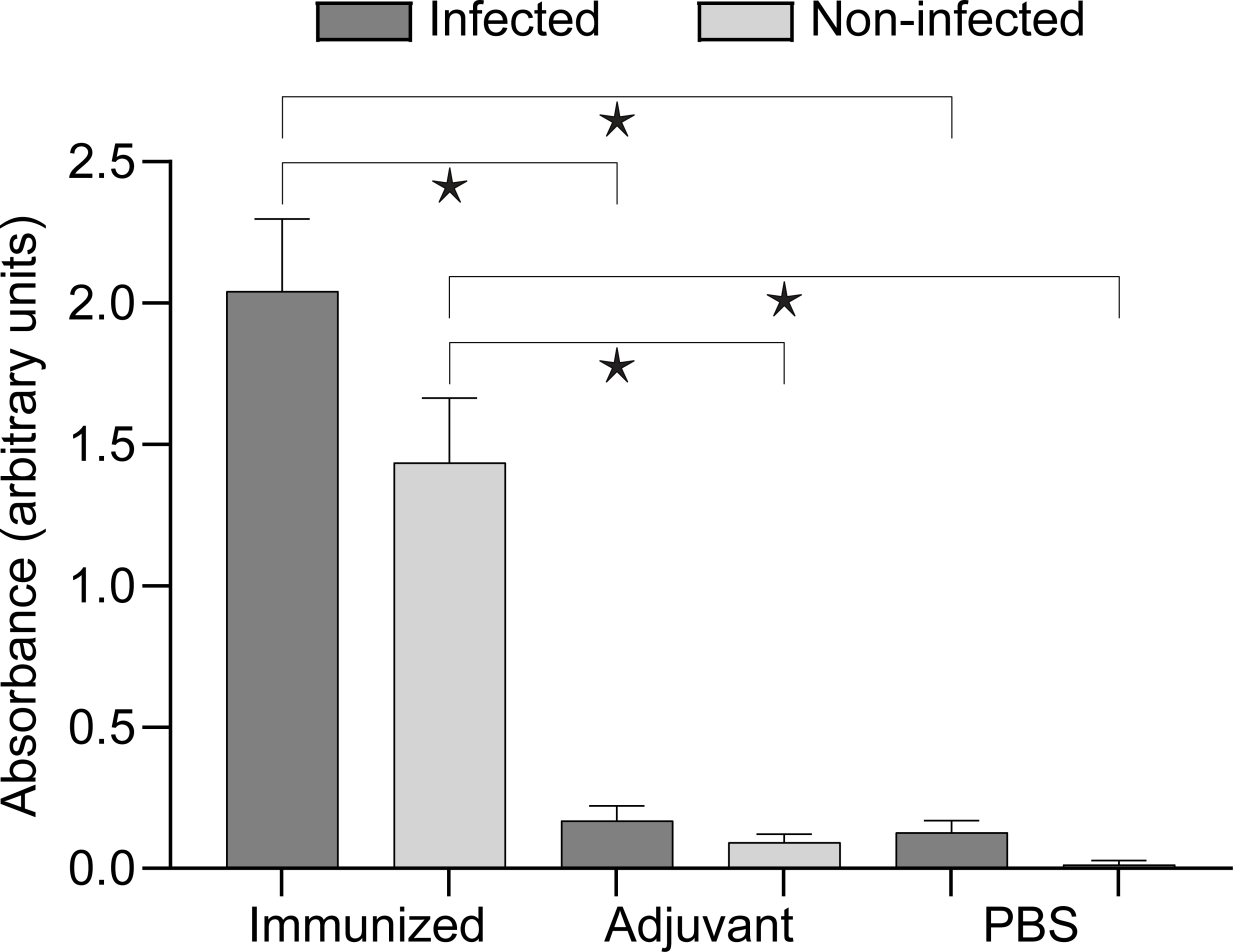
Antibody response of the experimental groups assessed by ELISA. Brackets with asterisks indicate significant differences (Kruskal–Wallis with Dunn’s multiple comparisons test, *P<0.001*). Detailed data for comparisons and their p values are presented in **Supplementary Table 2**.

Antibody responses as assessed by ELISA were significantly (Kruskal- Wallis test, *P<0.001*) elevated in both immunized groups (infected and non-infected) compared to the other groups. (**Figure 2**, **Supplementary Table 2**).

### 3.4 Morphological identification

All nematode larvae were identified to the genus level as *Anisakis* by their morphology. Characteristics were the ventriculus without appendage, absence of an intestinal caecum, presence of larval tooth, presence of mucron located at the posterior end, and the excretory pore placed anteriorly to nerve ring (**Supplementary Figure 1**).

### 3.5 Molecular identification

The ten sequences including partial 18S, complete ITS1, 5.8S, ITS2, and partial 28S were identical except for one specimen (GenBank acc. No PV696447) having two heterozygote nucleotides (both C→Y). These sequences (GenBank acc. nos. PV696447 to PV696456) were upon BLAST search at NCBI 99.79% found identical to PQ108495 from a sample of Atlantic herring *Clupea harengus* from the Danish part of the North Sea. The ten sequences of the partial *cox2* gene (PV711120 to PV711129) represented seven variants. However, in all cases the SNPs were neutral, as the resultant amino acid sequences were identical. BLAST search at NCBI excluding the primer binding sites resulted in a series of *A. simplex* sensu stricto entries having nucleotide identities from 99.83 to 100% (**Table 1**). The identification of the nematodes as *A. simplex* were confirmed by phylogenetic analysis of the ITS-region (excluding 18S and 28S) and of part of the *cox2* gene (**Supplementary Figure 2**).

**Table 1:** Identities of the partial *cox2* sequences of this study towards GenBank entries.

GenBank accession no.		Geographic location	Identity
This study	BLAST result		
PV711126	MN877346	Atlantic Northeast: FAO Zone 27^a^	99.83%
PV711127	MN877346	Atlantic Northeast: FAO Zone 27^a^	100%
PV711120	PP203000	Denmark: North Sea	100%
PV711121	PP203000	Denmark: North Sea	100%
PV711122	PP203000	Denmark: North Sea	99.83%
PV711125	PP203000	Denmark: North Sea	100%
PV711129	PQ126423	Denmark: North Sea	100%
PV711124	PQ126432	Denmark: North Sea	99.83%
PV711128	KT852524	Greenland	100%
PV711123	KT852467	Tampen: northern North Sea	100%

The sequence between the forward and reverse PCR primer were subjected to BLAST at GenBank. For each specimen only one of the entries exhibiting highest identity is shown.

### 3.6 SDS-PAGE

**Figure 3:**
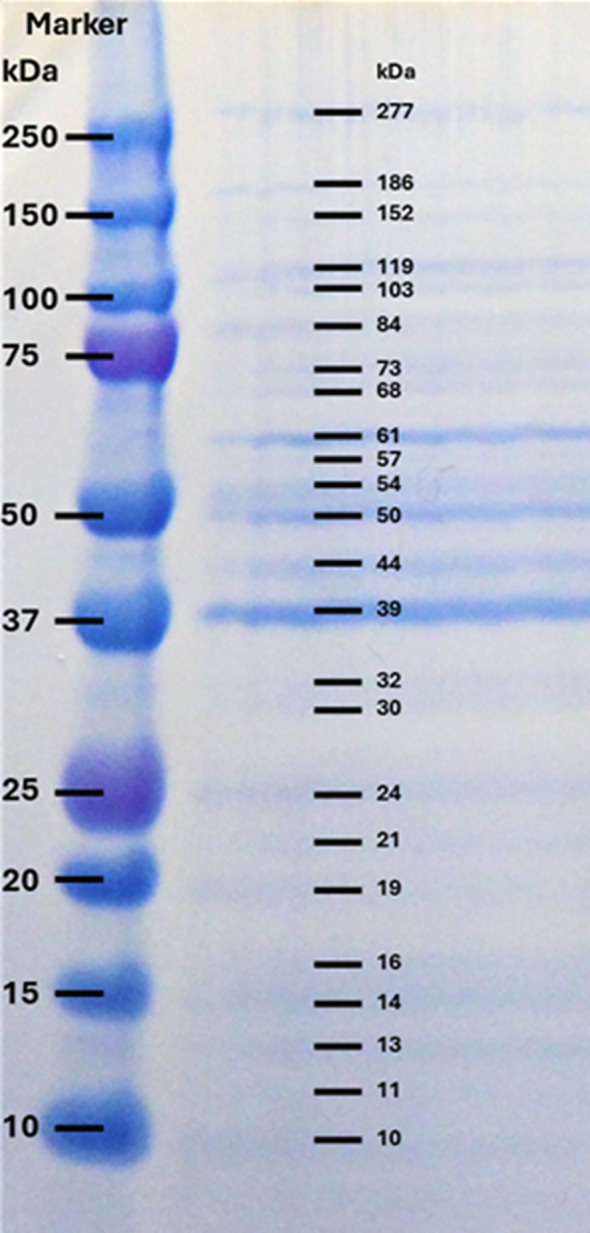
SDS-PAGE profile of crude extracts of *A. simplex* L3 under reducing condition, stained with SimplyBlue. Left column: Precision Plus Protein Dual Color Standards (BIO-RAD); Right column: *A. simplex* proteins with different molecular weights.

The SDS-PAGE profile of proteins in the immunogen preparation (supernatant of the *A. simplex* L3 homogenate) showed various protein bands with MWs 10, 11, 13, 14, 16, 19, 21, 24, 30, 32, 39, 44, 50, 54, 57, 61, 68, 73, 84, 103, 119, 152, 186, 277 kDa. Among these bands the highest staining intensities were observed at 39, 50 and 61 kDa (**Figure 3**).

### 3.7 Western blot

**Figure 4:**
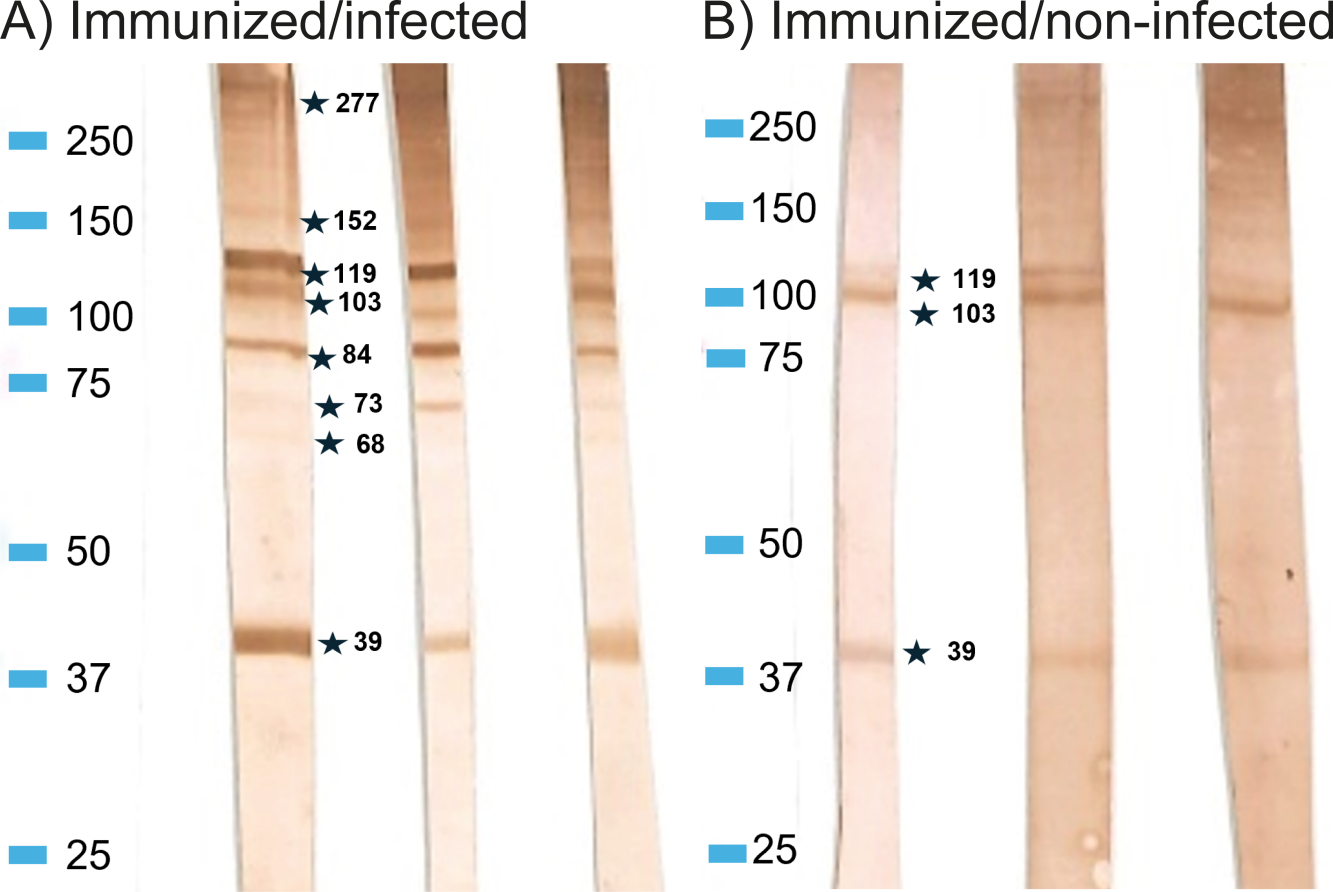
Western blot profiles showing reaction to (*A*) *simplex* L3 antigens of rainbow trout IgM of immunized/infected and immunized/non-infected fish. **(A)** Representative samples from Immunized/infected group, **(B)** Representative samples from Immunized/non-infected group. Marker: Precision Plus Protein Dual Color Standards (BIO-RAD).

Antibodies in plasma of both immunized/non-infected and immunized/infected rainbow trout reacted with a range of *A. simplex* proteins (**Figure 4**). However, plasma from adjuvant/non-infected and adjuvant/infected and PBS/non-infected and PBS/infected fish showed no reaction. Immunized/non-infected fish reacted with three proteins (MW 39, 103 and 119 kDa), while plasma from immunized/infected fish reacted with a higher number of antigens (39, 61, 73, 84, 103, 119, 152, 186 and 277 kDa). The strength of staining in both groups were graded on a scale from 1 to 3: weak (1), moderate (2), strong (3). In the immunized/infected group, the mean numbers of positive bands and their strength for each fish were 8.35 and 15.6, respectively. In the immunized/non-infected group the mean number of bands and their strength were 5.1 and 8.28, respectively. When comparing ELISA and Western-Blot results by the two-tailed Spearman Rank Correlation test we found a positive correlation between ELISA OD and WB band strength in both immunized/non-infected (*r= 0.6964; P<0.001*) and immunized/infected groups (*r= 0.7908*; *P<0.0001*).

### 3.8 Immune gene expression

**Figure 5:**
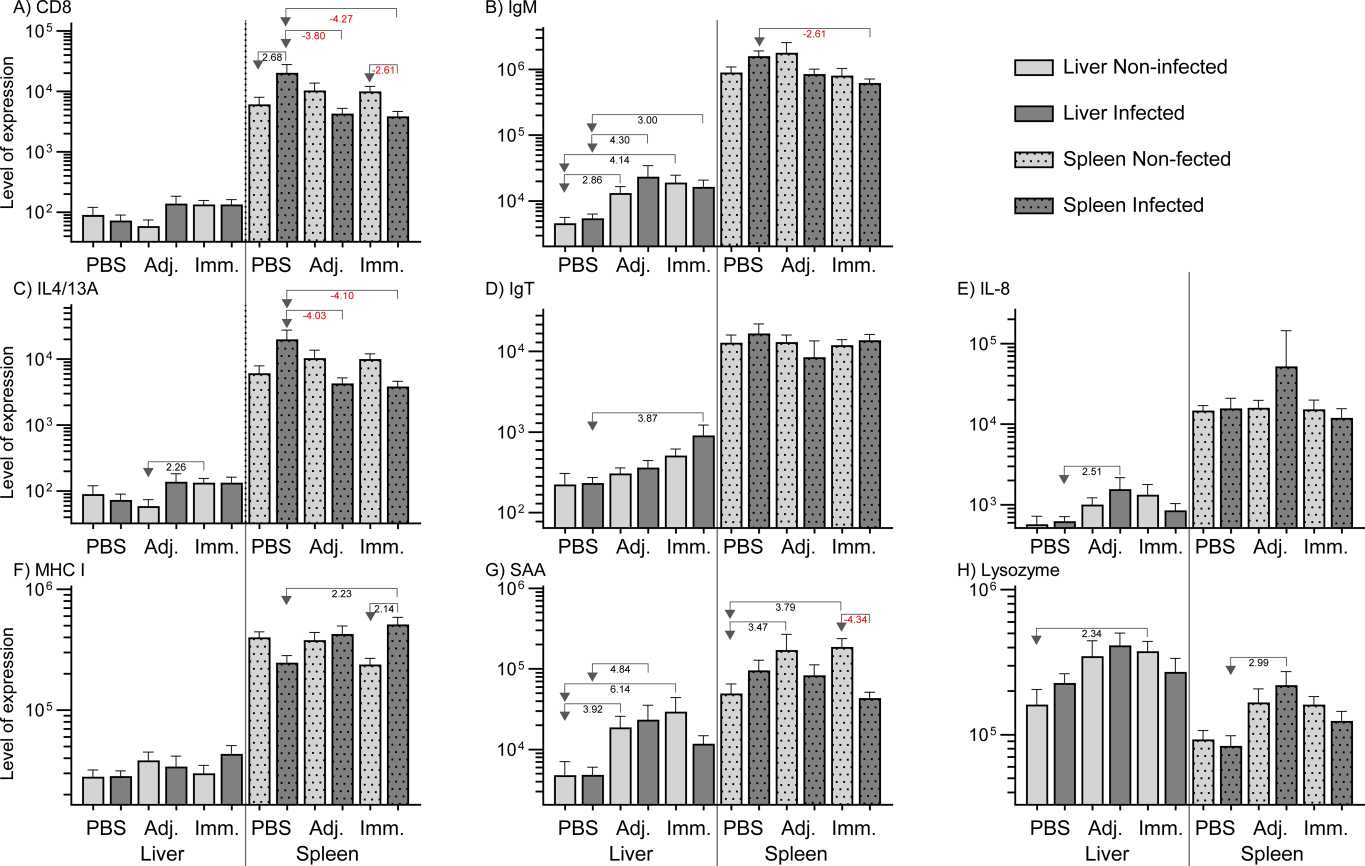
Relative expression of genes (high level expression). The levels were calculated as 2^-ΔCq^ divided by the level of the lowest expressed group (IL-22, immunized/non-infected). Significant folds (2^-ΔΔCq^) between two groups are indicated under arrows pointing from group of interest to reference group.

**Figure 6:**
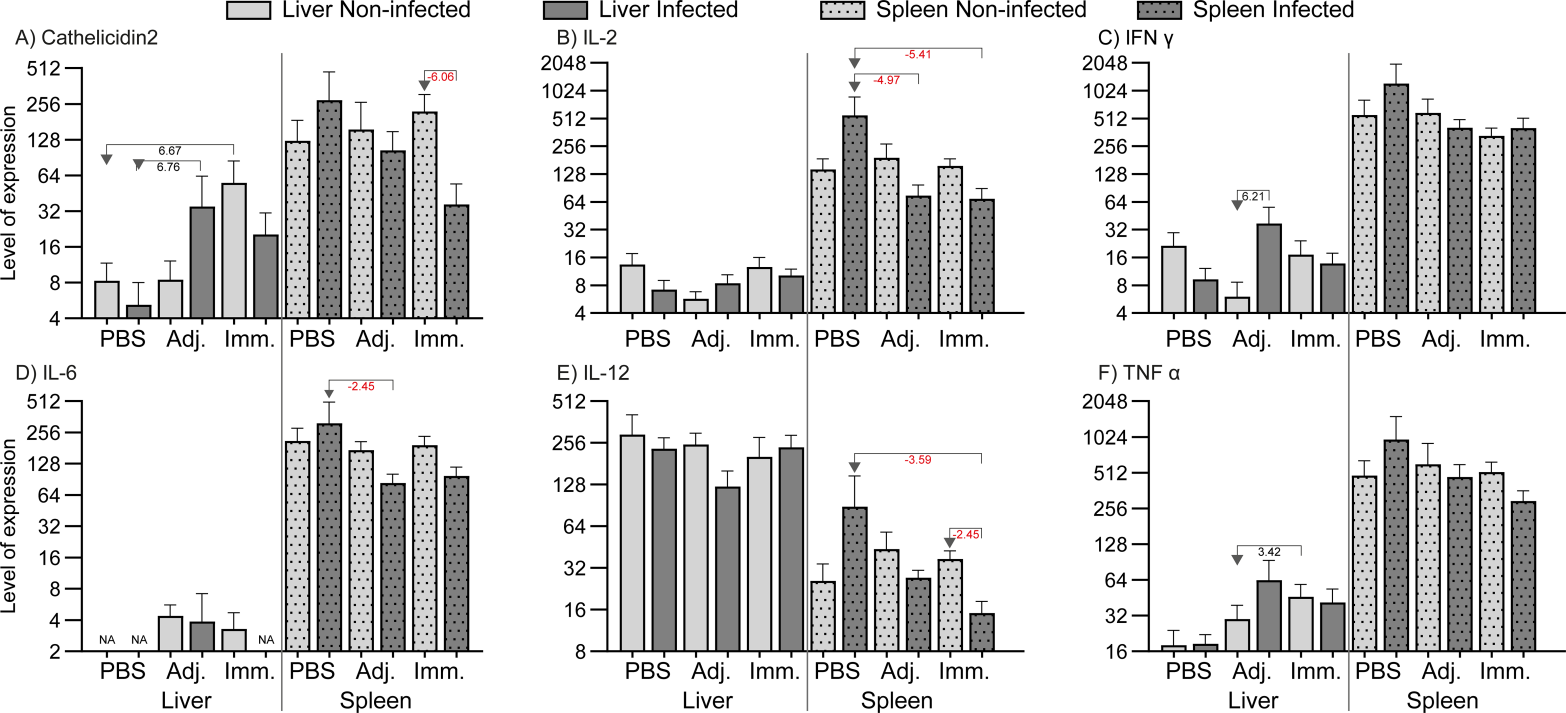
Relative expression of genes (low level expression). The levels were calculated as 2^-ΔCq^ divided by the level of the lowest expressed group (IL-22, immunized/non-infected). Significant folds (2^-ΔΔCq^) between two groups are indicated under arrows pointing from group of interest to reference group.

Immunization and infection induced some regulation of immune-relevant genes in fish organs (liver and spleen). **Supplementary File (Excel 1)** describes the qPCR result in detail. Genes with significant regulations (*P<0.05* and fold change at least ± 2) are presented as levels in **Figure 5** (high expression levels) and in **Figure 6** (low expression levels). In the liver genes encoding inflammatory cytokines (IFNγ, TNFα, IL-4/13A, IL-8), immunoglobulins (IgM, IgT), and acute phase proteins (Cathelicidin 2, Lysozyme, SAA) were up-regulated. In the spleen it was found that a few immune-relevant genes (encoding MHC1 and lysozyme) were upregulated, whereas others were down-regulated, including genes encoding cytokines such as IL-2, IL4/13A, IL-6, IL-12 and some encoding effector molecules such as IgM and Cathelicidin 2. No clear trend was noted for genes encoding CD8 and SAA.

## 4 Discussion

In the present study, we showed that immunization of rainbow trout with *A. simplex* L3 antigens elicited a measurable and marked specific IgM antibody response in the fish towards the nematode molecules. This was confirmed by ELISA and WB, whereas control and adjuvant-treated fish did not. In addition, prior immunization with worm antigens allowed rainbow trout to raise a partially protective response against live parasites, when compared to control fish. Fish injected only with adjuvant and PBS did not display a significant specific antibody production, even after exposure to live worms. This suggests that a single *A. simplex* infection over 25 d is insufficient to elicit a strong humoral response in naive rainbow trout. In addition, worm invasion alone did not induce macrophage aggregations (indicating a marked cellular reaction) in fish, which were merely treated with PBS. The fish immune system has been extensively studied during the latest three decades. Our knowledge on how salmonids reacts to invasive microorganisms (bacteria and virus) has been markedly expanded. A remarkable similarity to immune reactions in mammals has been documented. The initial reaction is an inflammatory reaction, which is followed by recruitment of innate response elements. However, at the same time a series of adaptive elements are activated, which lead to T and B cell associated reactions including production of a broad antibody repertoire within a few weeks (depend on temperature). The present study on the reaction of rainbow trout towards *A. simplex* infection shows a surprising lack of reaction in a central immune organ as the spleen (26, 30). One possible explanation is that the parasite employs immune evasion or immunosuppressive strategies that impair immune responses (2, 58). The presence of antigen-specific antibodies in the immunogen injected fish indicates that they engage in the adaptive immune system, including antigen recognition, presentation, B cell differentiation, and production of specific immunoglobulins. These pivotal elements bind to the invading pathogen resulting in subsequent neutralization, opsonization, complement activation, and increased phagocytic clearance (42, 43). Histopathological studies on *A. simplex* infected flatfish (44) and pelagic fish (45) documented that a series of immune cells, including neutrophils, are recruited to the invasion site in the fish. The present study confirmed that *A. simplex* located in the rainbow trout became encapsulated by host cells and among these melano-macrophages were detected. The presence of melano-macrophage aggregations in fish, injected with only adjuvant, indicates that these cells are part of an innate and non-specific response. However, the negative correlation between worm numbers and aggregations suggest that these cells, despite their non-specific nature, may play a role in protection. A marked adaptive reaction, reflected by an antibody production, was noted as well. Immunized trout (infected and non-infected) produced antibodies reacting with worm proteins (approximately MW 39, 103 and 119 kDa), but it was particularly noteworthy that fish immunized and subsequently infected with live larvae strongly reacted to those and additional antigens (approximately MW 61, 73, 84, 152, 186 and 277 kDa) suggesting that live larvae release additional antigens with a high immunogenic potential. In future studies aiming at pin-pointing the parasite molecules, which suppress the immune reaction in rainbow trout against *A. simplex* larvae, we will identify the antigens by use of proteomics (MS, amino acid composition). In the present study, aiming specifically to elucidate if prior immunization would elevate the antiparasitic response in trout, we limited the antigen characterization to MW determination of antigens detected in WB. Estimation of MW was here done, as in most studies, by use of SDS-PAGE, but it is not accurate and some variation may be found when comparing different studies. **Table 2** shows known *A. simplex* antigens (46), which correspond to a high extent to antigens detected in the present study. Thus (46), listed *A. simplex* proteins and it may therefore be speculated that our 39 kDa antigen could represent one or more of the *A. simplex* proteins with a MW of 40 kDa: Glyceraldehyde-3-P- dehydrogenase (Acc. no. P48812), Tropomyosin (Ani s 3) (Acc. No. Q9NAS5), Fructose-1,6- bisphosphatase (Ani s FBPP) (Acc. no. A8P3E5), Haemoglobin (Acces no. P26914), Malate dehydrogenase (Acc. no. F1L7C0), Arginine kinase (Acc. no. E1GBI0), 60S acidic ribosomal protein (Acc. no. A8PQF5), Antigenic IgI-domain (Acc. no. Q8MY16). The 73 kDA detected by our trout antibodies could correspond to the 70 kDa Heat shock protein 70 (Acc. no. A8Q5Z6) or the Phosphoenolpyruvate carboxykinase (Acc. no. Q05893). The 83 kDa antigen reacting with trout could be the 84 kDa Propionyl-CoAcarboxylase (Acc. no. F1KUZ6), Methylmalonyl-CoA mutase (Acc. no. F1KWB3), Heat shock protein 90 (Acc. no. C1KG49), 6-Phosphofructokinase (Acces no. F1KSL6), or Aconitate hydratase (Acces no. F1KYA7). The 103 kDa antigen found reactive in our rainbow trout could correspond to the 100 kDa antigen Pyruvate carboxylase 1 (Acces no. F1KRV7), Kinesin light chain (Acces no. Q05090), or the Calponin-like protein (Acces no. F1KPY3). The 186 kDa could be the 170kDa proteins RAS GTPase-activating protein (Acces no. F1KR99), Clathrin heavy chain (Acces no. F1KQ49), ATP-dependent RNA-helicase (Acces no. Q7QCW2), or the Coiled-coiled protein (Q7PQ25). The 277 kDa could represent: 200kDa: Myosin-4 (Acces no. F1KQ88), Filamin-A (Acces no. F1KPN0), Apolipophorin (F1KPM2), Carbonic anhydrase (Acces no. E0VS50). Several studies have listed these *A. simplex* proteins with corresponding molecular weights as allergens eliciting strong immune reactions in humans (46–49). Particularly low molecular weight proteins, ranging from 25 to 80 kDa, are associated with strong IgE-binding (46, 50). It is noteworthy that IgE in human patients bind strongly to *A. simplex* proteins of 37.7, 55.5 and 73.3 kDa MW proteins (46, 47). Some of these allergens may correspond to immunogens detected by rainbow trout.

**Table 2:** Candidate antigens of *Anisakis simplex* L3 corresponding to antigens reacting with rainbow trout plasma (this study).

kDa band in Western blot	Candidates	Accession no.
39	Glyceraldehyde-3-P- dehydrogenase	P48812 Q9NAS5 A8P3E5 P26914 F1L7C0 E1GBI0 A8PQF5 Q8MY16
	Tropomyosin (Ani s 3) Fructose-1,6- bisphosphatase (Ani s)	
	Haemoglobin Malate dehydrogenase	
	Arginine kinase 60S acidic ribosomal protein	
	Antigenic IgI-domain	
61	None	
73	70 kDa Heat shock protein 70 Phosphoenolpyruvate carboxykinase	A8Q5Z6 Q05893
84	Propionyl-CoAcarboxylase Methylmalonyl-CoA mutase Heat shock protein 90 6-Phosphofructokinase Aconitate hydratase	F1KUZ6 F1KWB3 C1KG49 F1KSL6 F1KYA7
103	Pyruvate carboxylase 1 Kinesin light chain Calponin-like protein	F1KRV7 Q05090 F1KPY3
119	None	
152	None	
186	RAS GTPase-activating protein Clathrin heavy chain ATP-dependent RNA-helicase the Coiled-coiled protein	F1KR99 F1KQ49 Q7QCW2 Q7PQ25
277	Myosin-4 Filamin-A Apolipophorin Carbonic anhydrase	F1KQ88 F1KPN0 F1KPM2 E0VS50

The immune gene expression analyses indicated a differential response of livers and spleens in the fish. In general, genes in the spleen were downregulated and genes in liver upregulated, indicating a differential involvement of immune organs in the reaction to the worm. Thus, immunized fish exhibited an upregulation of markers associated with a Th2-like profile, including the IgM encoding gene in the liver. Several key Th1 and Th2 pathway cytokines were downregulated in infected individuals across all groups, implying that *A. simplex* may induce broad immunosuppressive effects. Upregulation of the IFN-γ encoding gene in the adjuvant control group indicated a Th1-type response. With regard to innate immune genes, *cathelicidin 2*, *lysozyme*, and *saa* were upregulated in the liver of immunized fish without live nematode infection. This would indicate a general innate immune activation following *A. simplex* immunogen injection. Cathelicidins and lysozyme disrupt microbial membranes, while SAA functions as an opsonizer, enhancing phagocytosis by promoting the recognition and uptake of pathogens by phagocytic cells (51, 52). In addition, the gene encoding IFN-γ, a hallmark Th1 cytokine in mammals (53), was significantly upregulated in the liver of adjuvant-infected fish compared to uninfected adjuvant fish, suggesting that *A. simplex* infection can induce a Th1-type response under certain conditions. In contrast, expression of the *tnf-α* gene showed only a moderate increase in the liver of adjuvant-injected and subsequently infected fish relative to controls. This response likely reflects a nonspecific innate activation, as mineral oil-based adjuvants (such as the one used in this study) are known to trigger pro-inflammatory cytokine genes, including *tnf-α*, independently of antigen-specific stimulation (54). Reactions in the spleen differed. Genes encoding SAA and cathelicidin 2 were both downregulated in the spleen of infected immunized fish, despite prior priming, compared to the immunized non-infected. Further, in the immunized-infected group, the downregulation of genes encoding IL-2, IL-6, and IL-12, cytokines associated with T cell proliferation and Th1-like differentiation, indicates that *A. simplex* infection modulates key immune pathways in rainbow trout, particularly within the spleen. The study showed that 90% of *A. simplex* larvae were located in the body cavity, with only 10% in the musculature, a feature which may have implications for the host response (2). The immunogen preparation from sonicated larvae contains a heterogeneous mix of structural proteins, excretory/secretory (ES) products, and possibly potentially immunosuppressive molecules. It is likely that the immune response in rainbow trout requires a longer period to develop, particularly given the inherent difficulty of eliminating helminths due to their large size, complex structure, and evolved immune evasion mechanisms (3). Field studies on Atlantic mackerel showed that *A. simplex* infection levels may decrease with size and age (5), which suggests that immunity, as the type we demonstrate here, affects larval survival in fish. As noted above, anatomical distribution revealed that 90% of the recovered *Anisakis* larvae were located in the visceral organs, whereas only 10% were found in the musculature. This distribution aligns with previous studies indicating that *A. simplex* larvae typically penetrate the stomach wall and settle in the body cavity, particularly near the pyloric caeca and other visceral tissues (22, 55). Although the mechanisms driving this tissue tropism are not fully understood, it is likely that the viscera provide a more favorable environment for larval survival and encapsulation. However, *in vitro* studies (27) showed that *A. simplex* larvae react and coil up immediately after exposure to rainbow trout leukocytes, cells which are prevalent in the fish body cavity. This may explain that worm larvae coil up and remain in the host body cavity and refrain from further migration into the musculature. In line with this view, we also observed that the worm larvae were covered with host cells and, as part of these, melano-macrophage patches appeared on larvae and organs. The presence of these cells was exclusively recorded in the adjuvant- and immunized-infected groups, and their absence in PBS injected fish – even following an active infection - suggests that a protective immune priming can be partly based on these cell types. The negative correlation observed between infection intensity and melano-macrophage center (MMC) levels within the immunized group may indicate that fish with more prominent MMC responses harbor fewer parasites. A primary infection (3 weeks duration) alone appears to be insufficient to induce this response, but our experimental immunization may have accelerated a reaction, which takes longer time in previously non-exposed fish. Thus, under natural conditions MMCs serve as markers of chronic or prolonged immune stimulation in wild fish (56, 57). Alternatively, it could be suggested that *A. simplex* actively suppress macrophage activation, corresponding to the local immune depression found in liver of the Baltic cod, infected with *C. osculatum*, a related anisakid nematode larvae (58).

Apart from the humoral host IgM response, measured by ELISA and WB, we documented regulation of immune gene expression in rainbow trout in a organ-specific manner. This reflected distinct roles of the liver and spleen during *A. simplex* infection. In particular, the liver consistently showed upregulation of key immune markers in pre-treated groups, suggesting a strong activation of both innate and adaptive pathways. This observation is consistent with the general understanding that the teleost liver serves as an immunologically active organ, believed to host various immune cell populations, including IgM^+^ B cells (59). The spleen, as a secondary lymphoid organ, supports lymphocyte activation, antigen filtration, and immune memory, serving as a key site for mature B and T cells (60). Thus, in this work, the spleen frequently displayed downregulation of immune genes, particularly in infected fish, pointing to a potential suppressive effect or immune redirection (33). It may then be hypothesized that the nematode larvae, located in the body cavity, frequently depress the immune reaction locally as shown for *C. osculatum* (58).

Despite the antibody response and the cellular encapsulation detected, the immunized trout was not fully protected against the live nematode invasion. Fewer parasites were recovered after challenge, but the fish were not fully free from worms. This partial protection offered by injection of *A. simplex* homogenate into rainbow trout, as shown in this study, corresponds to the relative protection documented in other vertebrates immunized with various fractions of worms. Pigs and *Ascaris suum* have been widely used as a vertebrate host model, and several studies have reported induction of a partial protection against infection with *A. suum* larvae following injection of worm homogenates (12, 13). Some antigens may have higher effect than others, and it was reported that a recombinant version of a single antigen, worm enolase (also present in *A. simplex*), induced significant protection (12). These previous studies were performed by primary immunization followed by booster injections. This may suggest that future studies on immunization of trout against *A. simplex* can be optimized by use of one to several booster immunizations. In addition, the worm compounds released (E/S antigens) are believed to modulate immunity, and it would therefore be relevant to use these as immunogens in future studies. They are merely present in minor amounts in somatic homogenates of the worms, but they can be isolated in higher amounts following *in vitro* culture of the parasites (33, 47). In addition, it may be worthwhile to consider worm extracellular vesicles and their contents (61) as immunogens. Thus, it is likely that optimization of vaccines can be reached through the targeting of immunomodulatory molecules.

## 5 Conclusion

Rainbow trout may develop a partial antiparasitic response following immunization with *A. simplex* homogenate. The infection success, following oral administration of live *Anisakis* larvae, was significantly reduced in immunized trout compared to non-immunized fish (injected with PBS or adjuvant only). Melano-macrophage aggregation in the body cavity was activated by injection of immunogen and adjuvant and may have been associated with protection. Immunized fish (both trout immunized and subsequently infected by live worms and immunized fish without challenge) exhibited a marked high titer IgM response (ELISA, WB) towards *A. simplex* antigens. Innate and adaptive immune related genes were affected by adjuvant and immunogen injection and infection. Genes in spleen were markedly downregulated by immunization and infection, whereas some upregulation was noted in liver. Infection alone did not elicit a measurable antibody response over 25 d, and we hypothesize that the worm larvae secreted immunosuppressive substances. For future immunization studies we suggest including ES-antigens from *A. simplex* L3 in immunogen preparations. For this purpose the immune-regulating parasite molecules must be characterized by proteomic techniques (MS, amino acid sequencing). We suggest that this will target and neutralize immune regulating worm molecules, and thereby increase the protective response following infection with live parasites. This will further increase our understanding of mechanisms in parasites modulating host immune responses for increased survival of parasites in host animals.

## Data Availability

The original contributions presented in the study are included in the article/**Supplementary Material**, further inquiries can be directed to the corresponding author/s.

## Supplementary material

Supplementary Excel 1 Gene expression analyses. Fold regulations.

Supplementary Figure 1 Morphological characteristics discriminating *Anisakis simplex* third stage larvae.

Supplementary Figure 2 Phylograms of the ITS region (rDNA) and the mitochondrial gene *cox2*. Bootstrap value below 50% have been deleted.
