# Supplementary figures and images for: Protective immune response in rainbow trout (Oncorhynchus mykiss) against the parasitic nematode Anisakis simplex
Source: Front Immunol. 2025 Aug 20;16:1646450. doi: 10.3389/fimmu.2025.1646450 (PMC12404961; doi:10.3389/fimmu.2025.1646450)

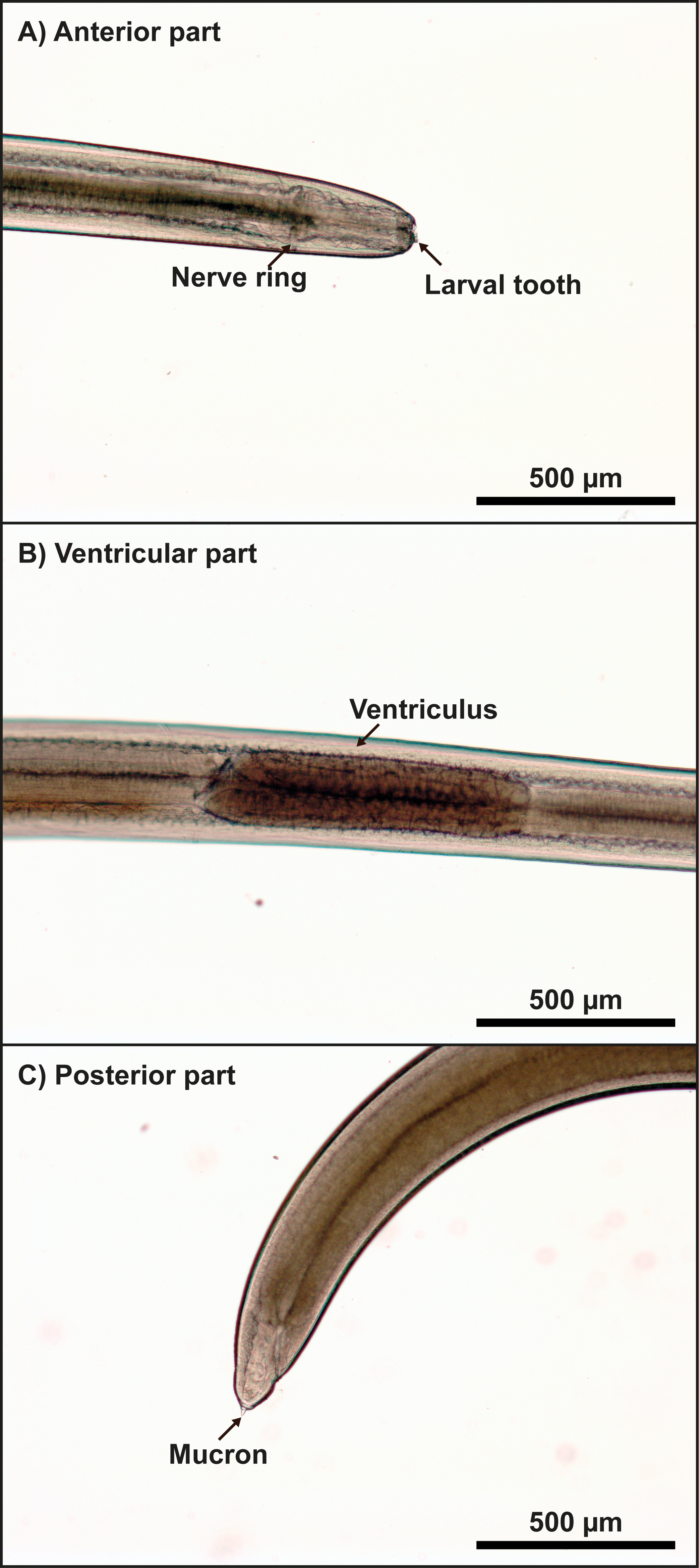

Supplement: Supplementary Figure 1 — Morphological characteristics discriminating Anisakis simplex third stage larvae. [file Image1.tif]
