# Supplementary material for: Protective immune response in rainbow trout (Oncorhynchus mykiss) against the parasitic nematode Anisakis simplex
Source: Front Immunol. 2025 Aug 20;16:1646450. doi: 10.3389/fimmu.2025.1646450 (PMC12404961; doi:10.3389/fimmu.2025.1646450)
Supplement: Supplementary file 4 [file Table1.docx]

**Supplementary Table 1.** Primers and probes used for qPCR assays. All nucleotides are from 5’ end (labeled with FAM) to 3’ end (labeled with BHQ1). All the qPCR assays were optimized to have annealing temperature of 60°C and having efficiencies of 100% ± 5%. ^R^ indicates reference genes (housekeepers). ^MS^ indicates the qPCR assay targets both membrane bound and secreted forms. ^1^ The α chain of IL-12 is common to the two isoforms of IL-12.

| **Gene**  **GenBank acc.no.** | **Length**  **Bp** | **Primers**  **5’end to 3’end** | **Probes**  **5’end to 3’end** | **References** |
| --- | --- | --- | --- | --- |
| ^R^ *arp*  AY505012 | 106 | Fwd: GAAAATCATCCAATTGCTGGATG  Rev: CTTCCCACGCAAGGACAGA | CTATCCCAAATGTTTCATTGTCGGCGC | [1] |
| ^R^ *β-actin*  AB196465 | 241 | Fwd: ACATCAAGGAGAAGCTGTGCTAC  Rev: TACGGATGTCCACGTCACAC | CCTCTCTGGAGAAGAGCTACGAGCTG | [2]  Probe [3] |
| ^R^ *elf-1α*  [AF498320](http://www.ncbi.nlm.nih.gov/entrez/viewer.fcgi?db=nucleotide&val=20269865) | 63 | Fwd: ACCCTCCTCTTGGTCGTTTC  Rev: TGATGACACCAACAGCAACA | GCTGTGCGTGACATGAGGCA | [4] |
| *c3.3* & *c3.4*  AF271080 / U61753 | 85 | Fwd: ATTGGCCTGTCCAAAACACA  Rev: AGCTTCAGATCAAGGAAGAAGTTC | TGGAATCTGTGTGTCTGAACCCC | [5] |
| *cathelicidin 1a*  AY382478 | 189 | Fwd: TCTCTCGTCCTGGGGTT  Rev: GTTGTAGCGTGCTGATCTATG | TAATTGGTCGTCCTGGGGGTGG | [3] |
| *cathelicidin 2a*  AY360356 | 135 | Fwd: AAAGATTCCAAGGGGGGT  Rev: CAAAGGGTGTGTTGTGCTGT | GCTCTCGTCCTGGGTTTGGCTCC | [6] |
| *cd4*  AY973028 | 89 | Fwd: CATTAGCCTGGGTGGTCAAT  Rev: CCCTTTCTTTGACAGGGAGA | CAGAAGAGAGAGCTGGATGTCTCCG | [7] |
| *cd8*  AF178054 | 74 | Fwd: ACACCAATGACCACAACCATAGAG  Rev: GGGTCCACCTTTCCCACTTT | ACCAGCTCTACAACTGCCAAGTCGTGC | [8] |
| *ifn γ1* and i*fn γ2*  FJ184374 / FJ184375 | 68 | Fwd: AAGGGCTGTGATGTGTTTCTG  Rev: TGTACTGAGCGGCATTACTCC | TTGATGGGCTGGATGACTTTAGGA | [9] |
| *igdm*  AY870262 | 304 | Fwd: CAGGAGGAAAGTTCGGCATCA  Rev: CCTCAAGGAGCTCTGGTTTGGA | CCACACCACACAGACTCTGGCCCTGAA | [10] |
| *igds*  JQ003979 | 304 | Fwd: TGGCACGCCAGGATTTGAC  Rev: TCAGAATTGAGTGAACGGACAGACA | CCACACCACACAGACTCTGGCCCTGAA | [10] |
| ^MS^ *IgM*  S63348 / AH014877 | 72 | Fwd: ACCCTCCTCTTGGTCGTTTC  Rev: TGATGACACCAACAGCAACA | TGATGACACCAACAGCAACA | [9] |
| ^MS^ *igt*  AY870265 / AY870263 | 73 | Fwd: AGCACCAGGGTGAAACCA  Rev: GCGGTGGGTTCAGAGTCA | AGCAAGACGACCTCCAAAACAGAAC | [9] |
| *IL1 ß*  AJ223954 | 91 | Fwd: ACATTGCCAACCTCATCATCG  Rev: TTGAGCAGGTCCTTGTCCTTG | CATGGAGAGGTTAAAGGGTGGC | [9] |
| *iL2a*  FJ571513 | 110 | Fwd: ATGCAACACCACATCAGCAT  Rev: TGCCACGGCCCTACAAAAGA  RE  TGCCACGGCCCTACAAAAGA | TGCCACGGCCCTACAAAAGA | [3] |
| *il-4/13a*  AB574337 | 138 | Fwd: ATCCTTCTCCTCTCTGTTGC  Rev: GAGTGTGTGTGTATTGTCCTG | CGCACCGGCAGCATAGAAGT | [11] |
| *il-6a*  DQ866150 | 91 | Fwd: ACTCCCCTCTGTCACACACC  Rev: *GGCAGACAGGTCCTCCACTA* | CCACTGTGCTGATAGGGCTGG | [12] |
| *il-8*  isoforms a, b , c, d & e  AY160982 to AY160986 | 69 | Fwd: AGAATGTCAGCCAGCCTTGT  Rev: TCTCAGACTCATCCCCTCAGT | TTGTGCTCCTGGCCCTCCTGA | [12] |
| *il-10*  [AB118099](http://www.ncbi.nlm.nih.gov/entrez/viewer.fcgi?db=nucleotide&val=47678892) | 70 | Fwd: CGACTTTAAATCTCCCATCGAC  Rev: GCATTGGACGATCTCTTTCTTC | CATCGGAAACATCTTCCACGAGCT | [9] |
| ^1^ *il-12α* chain  HE798148 | 84 | Fwd: CAACGGAACACCACATTCAG  Rev: AGCCTGTAGTGAGGCAGCAT | TGCGTGTCTGAGGAACATCCG | [13] |
| *il-17a/f2*  AJ580842 | 158 | Fwd: TCAAAAGCAACGTGTCGAAG  Rev: TCCCTCTGATTCCTCTGTGG | TATGCTGCTGGGCCTGACCA | [13] |
| *il-17c1*  CAW30792 | 138 | Fwd: CTGGCGGTACAGCATCGATA  Rev: GAGTTATATCCATAATCTTCGTATTCGGC | CGTGATGTCCGTGCCCTTTGACGATG | [11] |
| *il-17c2*  CAW30793 | 134 | Fwd: CTGGCGGTACAGCATCGATA  Rev: CAGAGTTATATGCATGATGTTGGGC | CGTGGTGTCCAGGCCCTTTAATGATG | [11] |
| *il-22*  AM748537 | 64 | Fwd: ATGACCACCACCACAGCATT  Rev: ATTCCTTTCCCCTCCTCCAT | CTTTCCGCAAGAAGTTGTCCGAG | [8] |
| *lysozyme*  X59491 | 188 | Fwd: GAAACAGCCTGCCCAACT  Rev: GTCCAACACCACACGCTT | ATACCCAGGCCACCAACCGCAACAC | [14] |
| *saa*  AM422446 | 79 | Fwd: GGGAGATGATTCAGGGTTCCA  Rev: TTACGTCCCCAGTGGTTAGC | TCGAGGACACGAGGACTCAGCA | [15] |
| *tcr-ß*  AF329700 | 73 | Fwd: TCACCAGCAGACTGAGAGTCC  Rev: AAGCTGACAATGCAGGTGAATC | CCAATGAATGGCACAAACCAGAGAA | [9] |
| *tgf-ß1*  [X99303](http://www.ncbi.nlm.nih.gov/entrez/viewer.fcgi?db=nucleotide&val=1478246) | 75 | Fwd: TCTGAATGAGTGGCTGCAAG  Rev: GGTTTCCCACAATCACAAGG | CTGGAGAGGAGCAGGGATTCCAAT | [9] |
| *tnf-α1* & *tnf-α2*  AJ277604 / AJ401377 | 75 | Fwd: GGGGACAAACTGTGGACTGA  Rev: GAAGTTCTTGCCCTGCTCTG | GACCAATCGACTGACCGACGTGGA | [12] |

[1] Purcell MK, Kurath G, Garver KA, Herwig RP, Winton JR. Quantitative expression profiling of immune response genes in rainbow trout following infectious haematopoietic necrosis virus (IHNV) infection or DNA vaccination. *Fish Shellfish Immunol*. (2004) 17:447-62. [doi: 10.1016/j.fsi.2004.04.017](https://doi.org/10.1016/j.fsi.2004.04.017)

[2] Sugiura SH, Kelsey K, Ferraris RP. Molecular and conventional responses of large rainbow trout to dietary phosphorus restriction. *J Comp Physiol B*. (2007) 177:461-72. [doi: 10.1007/s00360-007-0144-9](https://doi.org/10.1007/s00360-007-0144-9)

[3] Zuo S, Karami AM, Ødegård J, Mathiessen H, Marana MH, Jaafar RM, et al. Immune gene expression and genome-wide association analysis in rainbow trout with different resistance to *Yersinia ruckeri* infection. *Fish Shellfish Immuno*l. (2020) 106:441-50. [doi: 10.1016/j.fsi.2020.07.023](https://doi.org/10.1016/j.fsi.2020.07.023)

[4] Ingerslev HC, Pettersen EF, Jakobsen RA, Petersen CB, Wergeland HI. Expression profiling and validation of reference gene candidates in immune relevant tissues and cells from Atlantic salmon (*Salmo salar* L.). *Mol Immunol.* (2006) 43:1194-201. [doi: 10.1016/j.molimm.2005.07.009](https://doi.org/10.1016/j.molimm.2005.07.009)

[5] Raida MK, Buchmann K. Innate immune response in rainbow trout (*Oncorhynchus mykiss*) against primary and secondary infections with *Yersinia ruckeri* O1. *Dev Comp Immunol*. (2009) 33:35-45. [doi: 10.1016/j.dci.2008.07.001](https://doi.org/10.1016/j.dci.2008.07.001)

[6] Xueqin J, Kania PW, Buchmann K. Comparative effects of four feed types on white spot disease susceptibility and skin immune parameters in rainbow trout, *Oncorhynchus mykiss* (Walbaum). *J Fish Dis*. (2012) 35:127-35. [doi: 10.1111/j.1365-2761.2011.01329.x](https://doi.org/10.1111/j.1365-2761.2011.01329.x)

[7] Raida MK, Buchmann K. Development of adaptive immunity in rainbow trout, *Oncorhynchus mykiss* (Walbaum) surviving an infection with *Yersinia ruckeri*. *Fish Shellfish Immunol*. (2008) 25:533-41. [doi: 10.1016/j.fsi.2008.07.008](https://doi.org/10.1016/j.fsi.2008.07.008)

[8] Olsen MM, Kania PW, Heinecke RD, Skjoedt K, Rasmussen KJ, Buchmann K. Cellular and humoral factors involved in the response of rainbow trout gills to *Ichthyophthirius multifiliis* infections: molecular and immunohistochemical studies. *Fish Shellfish Immunol*. (2011) 30:859-69. [doi: 10.1016/j.fsi.2011.01.010](https://doi.org/10.1016/j.fsi.2011.01.010)

[9] Raida MK, Buchmann K. Temperature-dependent expression of immune-relevant genes in rainbow trout following *Yersinia ruckeri* vaccination. Dis. Aquat Organ. (2007) 77:41-52. [doi: 10.3354/dao01808](https://doi.org/10.3354/dao01808)

[10] Skov J, Chettri JK, Jaafar RM, Kania PW, Dalsgaard I, Buchmann K. Effects of soluble immunostimulants on mucosal immune responses in rainbow trout immersion-vaccinated against *Yersinia ruckeri*. *Aquaculture*. (2018) 492:237-46. [doi: 10.1016/j.aquaculture.2018.04.011](https://doi.org/10.1016/j.aquaculture.2018.04.011)

[11] Chettri JK, Kuhn JA, Jaafar RM, Kania PW, Møller OS, Buchmann K. Epidermal response of rainbow trout to *Ichthyobodo necator*: immunohistochemical and gene expression studies indicate a T h1‐/T h2‐like switch. *J Fish Dis*. (2014) 37:771-83. [doi: 10.1111/jfd.12169](https://doi.org/10.1111/jfd.12169)

[12] Raida MK, Buchmann K. Bath vaccination of rainbow trout (*Oncorhynchus mykiss* Walbaum) against *Yersinia ruckeri*: effects of temperature on protection and gene expression. *Vaccine*. 2008 26:1050-62. [doi: 10.1016/j.vaccine.2007.12.029](https://doi.org/10.1016/j.vaccine.2007.12.029)

[13] Jaafar RM, Chettri JK, Dalsgaard I, Al-Jubury A, Kania PW, Skov J, Buchmann K. Effects of adjuvant Montanide™ ISA 763 A VG in rainbow trout injection vaccinated against *Yersinia ruckeri*. *Fish Shellfish Immunol*. (2015) 47:797-806. [doi:10.1016/j.fsi.2015.10.023](https://doi.org/10.1016/j.fsi.2015.10.023)

[14] Chettri JK, Raida MK, Kania PW, Buchmann K. Differential immune response of rainbow trout (*Oncorhynchus mykiss*) at early developmental stages (larvae and fry) against the bacterial pathogen *Yersinia ruckeri*. *Dev Comp Immunol*. (2012) 36:463-74. [doi: 10.1016/j.dci.2011.08.014](https://doi.org/10.1016/j.dci.2011.08.014)

[15] Skov J, Kania PW, Holten-Andersen L, Fouz B, Buchmann K. Immunomodulatory effects of dietary β-1, 3-glucan from *Euglena gracilis* in rainbow trout (*Oncorhynchus mykiss*) immersion vaccinated against *Yersinia ruckeri*. Fish Shellfish Immunol. 2012 33:111-20. [doi: 10.1016/j.fsi.2012.04.009](https://doi.org/10.1016/j.fsi.2012.04.009)
