# Supplementary material for: Protective immune response in rainbow trout (Oncorhynchus mykiss) against the parasitic nematode Anisakis simplex
Source: Front Immunol. 2025 Aug 20;16:1646450. doi: 10.3389/fimmu.2025.1646450 (PMC12404961; doi:10.3389/fimmu.2025.1646450)
Supplement: Supplementary file 5 [file Table2.docx]

**Supplementary Table 2.**Comparison list and ELISA results. Numbers indicate significant difference (probability level P value), and ns indicate no significant difference between groups compared (Kruskal-Wallis with Dunn’s multiple comparisons test, P< 0.001).

| **Comparison List** | ***P values*** |
| --- | --- |
| PBS/non- infected vs. PBS/infected | ns |
| Adjuvant/non-infected vs. Adjuvant/infected | ns |
| Immunized/non-infected vs. Immunized/infected | ns |
| PBS/non-infected vs. Adjuvant/non-infected | ns |
| PBS/non-infected vs. Immunized/non infected | *<0.0001* |
| Adjuvant/non-infected vs. Immunized/non-infected | *0,0008* |
| PBS/infected vs. Adjuvant/infected | ns |
| PBS/infected vs. Immunized/infected | *<0.0001* |
| Adjuvant/infected vs. Immunized/infected | *<0.0001* |
